# Supplementary material for: Development of a Novel Enzyme-Linked Immunosorbent Assay and Lateral Flow Test System for Improved Serodiagnosis of Visceral Leishmaniasis in Different Areas of Endemicity
Source: Microbiol Spectr. 2023 Apr 19;11(3):e04338-22. doi: 10.1128/spectrum.04338-22 (PMC10269724; doi:10.1128/spectrum.04338-22)
Supplement: Supplemental file 1 — Supplemental material. Download spectrum.04338-22-s0001.pdf, PDF file, 1.1 MB [file spectrum.04338-22-s0001.pdf]

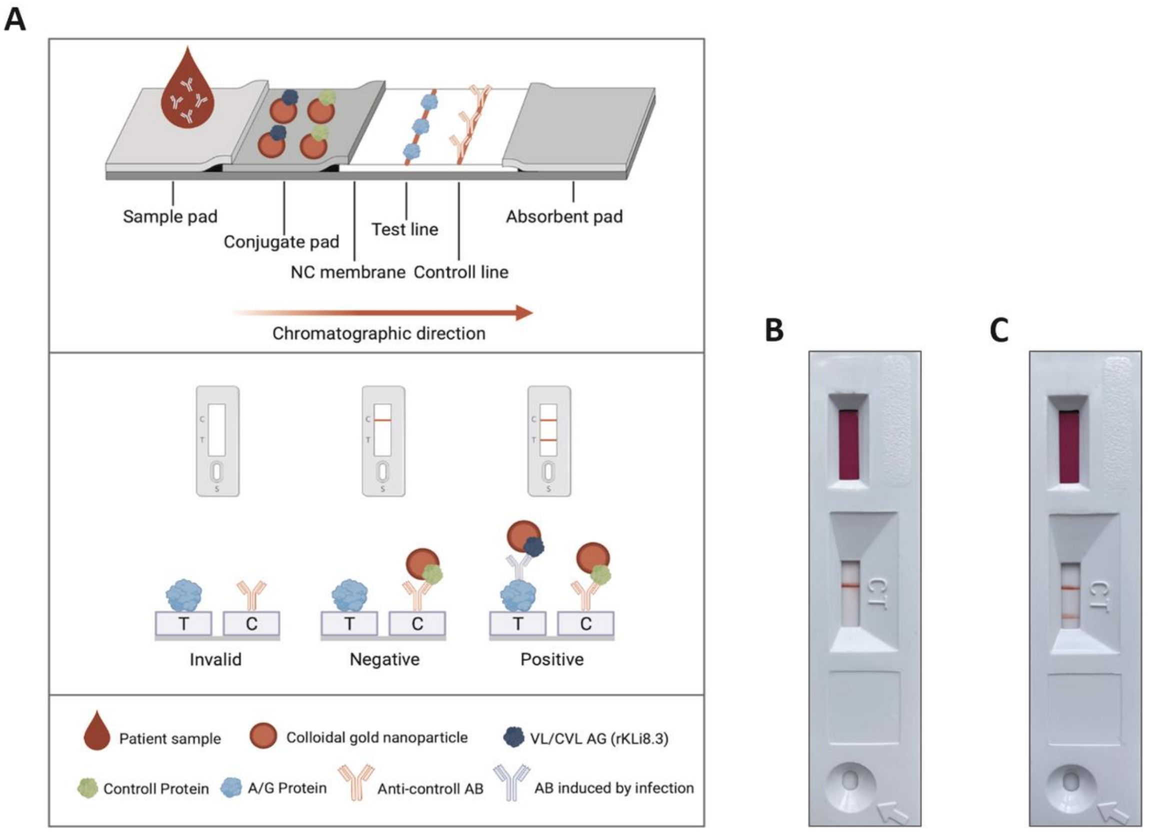

Supplemental Figure S1. Lateral flow test (LFT) for detection of VL specific antibodies. (**A**) Schematic assembly of LFT device. (**B**) Example of negative and (**C**) positive VL antibody reaction.

Supplemental Table S1. Kinesin AA diversity of different *Leishmania* strains from Sudan compared with published sequences of rK39, KE16 and rKLO8

	P	1	2	3	4	5	6	7	8	9	10	11	12	13	14	15	16	17	18	19	20	21	22	23	24	25	26	27	28	29	30	31	32	33	34	35	36	37	38	39
KE16	C	L	E	Q	R	L	R	D	S	E	E	R	A	A	E	L	K	R	K	L	E	A	T	A	A	A	K	S	S	A	E	Q	D	R	E	N	T	R	A	T
					Q			E			A	H					M	A	Q			S						T												A
																	A	S																						
rK39	C	L	E	Q	Q	L	R	D	S	E	E	R	A	A	E	L	A	S	Q	L	E	A	T	A	A	A	K	M	S	A	E	Q	D	R	E	N	T	R	A	T
								E			A											S		T				S								S				A
rKLO8	S	L	E	Q	Q	L	R	D	S	E	E	R	A	A	E	L	A	S	Q	L	E	A	T	A	A	A	K	M	S	A	E	Q	D	R	E	N	T	R	A	T
							L	E	L					G								S		T				S								S				A
																												T												
Sudanese *L. archibaldi*	S	L	E	Q	R	L	R	D	S	E	E	R	A	A	E	L	K	S	E	L	E	A	T	A	A	A	K	M	S	A	E	Q	D	R	E	N	T	R	A	T
					L		L	E			A			G			A	A	Q			S		T				S												A
					Q																							T												
Sudanese *L. donovani*	S	L	E	Q	Q	L	R	D	S	E	E	R	A	A	E	L	K	S	E	L	E	S	T	T	A	A	K	M	S	A	E	Q	D	R	E	N	T	R	A	T
					L		L	E			A			G			A	A	Q			A		A				S												A
																												T												
Sudanese *L. infantum*	S	L	E	Q	R	L	R	D	S	E	E	R	A	A	E	L	K	S	E	L	E	S	T	T	A	A	K	M	S	A	E	Q	D	R	E	N	T	R	A	T
					L		L	E			A			G			A	A	Q			A		A				S												A
					Q																							T												

P, Pre-Repeat Region.
Boxes indicate substitution of charged for uncharged AA.

Supplemental Table S2. Kinesin AA diversity of recombinant rKLi8.3, rKLd8.3 and rKLa8.3 antigens

	P	1	2	3	4	5	6	7	8	9	10	11	12	13	14	15	16	17	18	19	20	21	22	23	24	25	26	27	28	29	30	31	32	33	34	35	36	37	38	39
rKLi8.3	C	L	E	Q	Q	L	R	D	S	E	E	R	A	A	E	L	A	S	Q	L	E	A	T	A	A	A	K	M	S	A	E	Q	D	R	E	N	T	R	A	T
					L			E														S		T				S												A
																												T												
rKLd8.3	C	L	E	Q	Q	L	R	D	S	E	E	R	A	A	E	L	K	S	E	L	E	S	T	T	A	A	K	M	S	A	E	Q	D	R	E	N	T	R	A	T
					L			E			A						A	A	Q			A		A				S												A
																												T												
rKLa8.3	C	L	E	Q	Q	L	R	D	S	E	E	R	A	A	E	L	A	S	Q	L	E	S	T	T	A	A	K	M	S	A	E	Q	D	R	E	N	T	R	A	T
					L			E			A											A		A				S												A
																												T												

P, Pre-Repeat Region.
rKLi8.3: *L. infantum* with 8.3 tandem repeats.
rKLd8.3: *L. donovani* with 8.3 tandem repeats.
rKLa8.3: *L. archibaldi* with 8.3 tandem repeats.
Boxes indicate substitution of charged for uncharged AA.

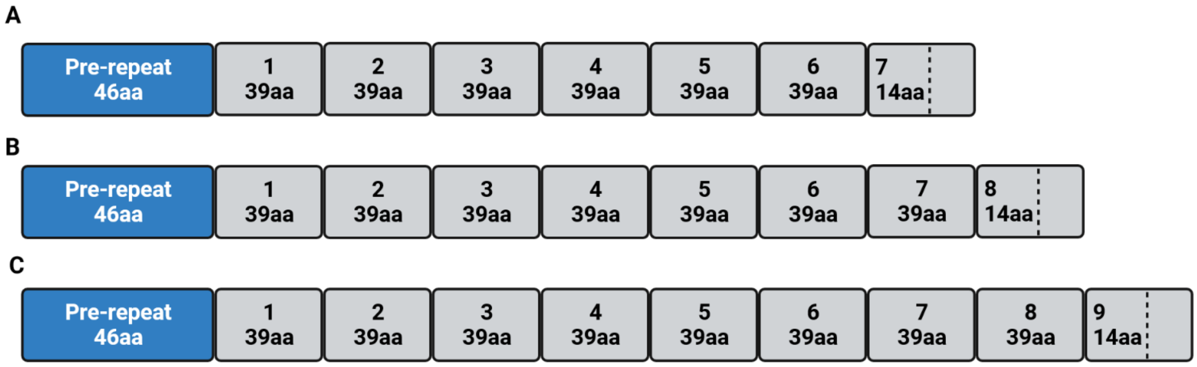

Supplemental Figure S2. Schematic representations of kinesin antigens with increasing numbers of tandem repeats. (**A**) rKLi6.3 (**B**) rKLi7.3 (**C**) rKLi8.3. The numbered gray boxes represents the complete (39 amino acids) or the partial (14 amino acids) repetitive motif units of kinesin. Pre-repeat region are shown in Blue.

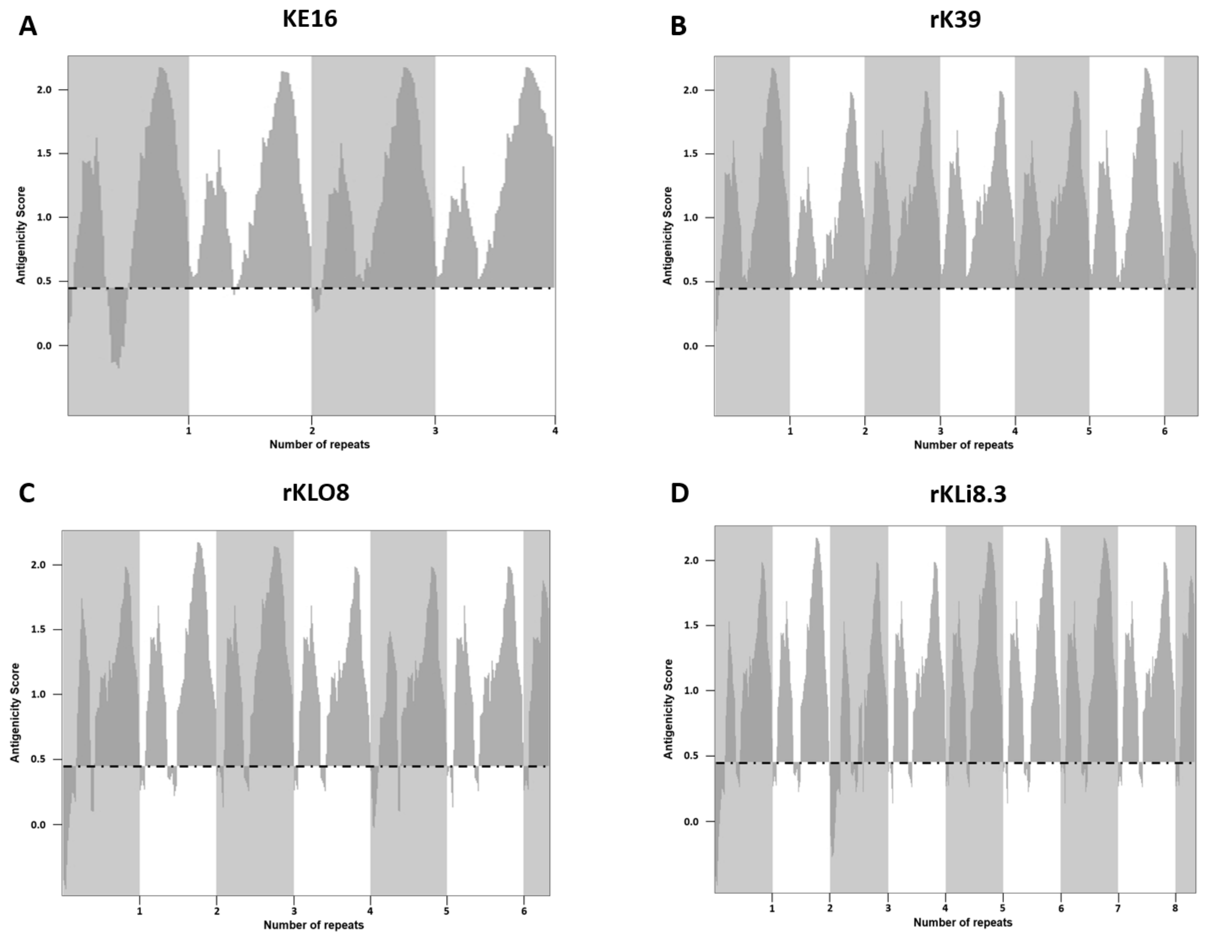

Supplemental Figure S3. Linear prediction of B-cell epitopes. The prediction was performed with BepiPred 1.0, with a cut-off = 0.48. Plots of antigenicity values were created in the programming environment R v. 3.5.2 using the package ggplot2 v. 3.3.2. Gray and white boxes represent tandem repeat motifs of 39 AA. The area under curve is for **(A)** 117.6, **(B)** 184.5, **(C)** 172.3 and **(D)** 222.2.

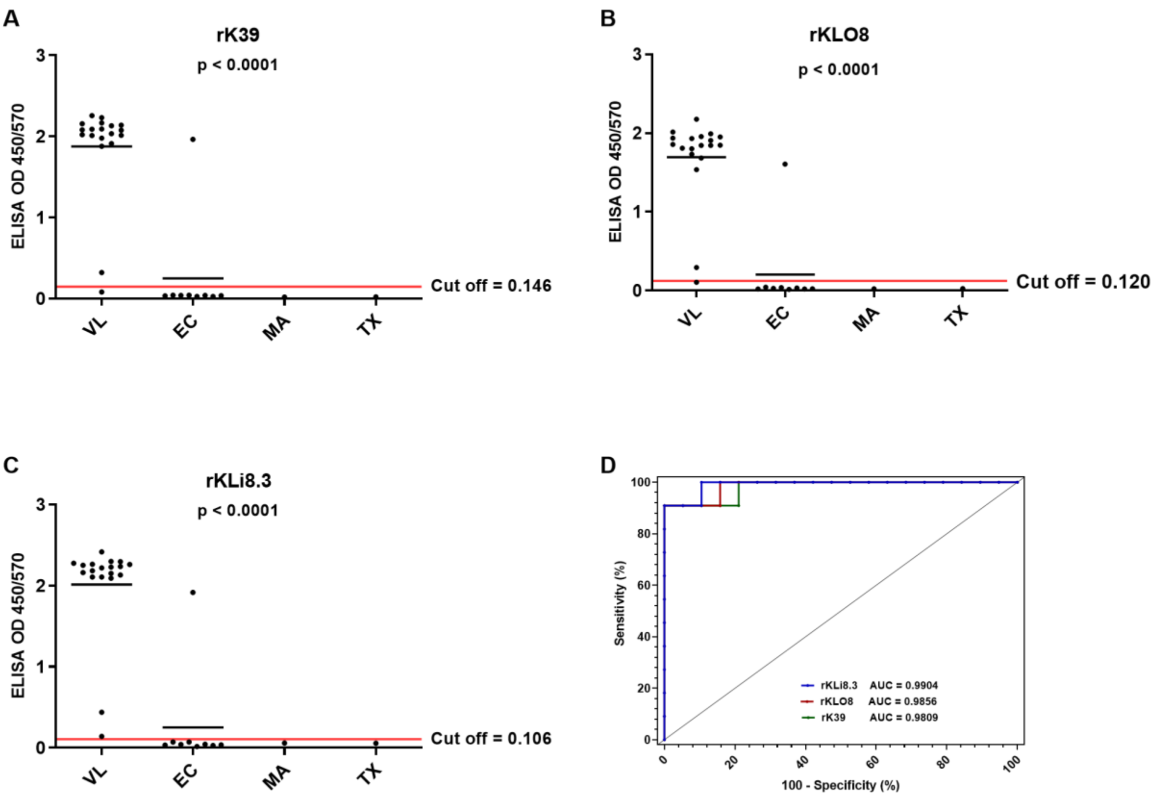

Supplemental Figure S4. Antibody responses of VL sera from India. (**A**) rK39-, (**B**) rKLO8-, and (**C**) rKLi8.3- based ELISA. VL [n = 19], endemic controls (EC) [n = 11], toxoplasmose (TX) [n = 1] and malaria (MA) [n = 1]. (**D**) ROC curves for rK39-, rKLO8- and rKLi8.3-ELISA.

Supplemental Table S3. VL-diagnostic performance of rK39, rKLO8 and rKLi8.3 ELISA in India

ELISA Test	Cut-off	AUC	TP	FN	TN	FP	Sensitivity (%)	Specificity (%)	PPV (%)	NPV (%)	DEV (%)
rK39	0.146	0.9809	18	1	10	1	94.73	90.91	94.73	90.91	93.33
rKLO8	0.120	0.9856	18	1	10	1	94.73	90.91	94.73	90.91	93.33
rKLi8.3	0.106	0.9904	19	0	10	1	100	90.91	95	100	96.66

AUC, area under curve; TP, true positive; FN, false negative; TN, true negative; FP, false positive; PPV, positive predictive value; NPV, negative predictive value; DEV, diagnostic efficiency value.
